# Clinical Factors Associated with Termination of Pregnancy Recommendations Following Prenatal Diagnosis of Congenital Heart Disease: A Multidisciplinary Council-Based Study

**DOI:** 10.3390/jcm15082838

**Published:** 2026-04-09

**Authors:** Ilayda Gercik Arzik, Hakan Golbasi, Zubeyde Emiralioglu Cakir, Hale Ankara Aktas, Bahar Konuralp Atakul, Didem Gul Saritas, Deniz Boz Eravci, Atalay Ekin

**Affiliations:** 1 Department of Perinatology, Izmir City Hospital, Izmir 35540, Turkey; drhkngolbasi@gmail.com (H.G.); haleankara@gmail.com (H.A.A.); baharkonuralp@gmail.com (B.K.A.); didemgul83@hotmail.com (D.G.S.); atalayekin@hotmail.com (A.E.); 2 Division of Perinatology, Department of Obstetrics and Gynecology, Buca Seyfi Demirsoy Education and Research Hospital, Izmir 35390, Turkey; zubeydeemiralioglu@hotmail.com; 3 Centre for Labor and Social Security Training and Research, Ministry of Labor and Social Security, Ankara 06510, Turkey; denizbozdb@gmail.com

**Keywords:** congenital heart disease, termination of pregnancy, prenatal diagnosis, multidisciplinary counseling

## Abstract

**Objective:** To evaluate clinical factors associated with termination of pregnancy (TOP) recommendations following prenatal diagnosis of congenital heart disease (CHD) within a multidisciplinary fetal council model. **Methods:** This retrospective cohort study included 146 fetuses with prenatally diagnosed CHD discussed in a tertiary referral center fetal council between October 2023 and December 2025. The primary outcome was council-issued recommendation for TOP (yes/no). Variables included gestational age (GA) at diagnosis, cardiac severity (Davey scale grouped as low, moderate, high), extracardiac anomalies, fetal growth restriction (FGR), and genetic evaluation/results. Group comparisons were performed using Mann–Whitney U and χ^2^ tests. Independent associations were assessed using binary logistic regression. A subgroup analysis was conducted in isolated CHD cases (no extracardiac structural anomalies). **Results:** A total of 146 fetuses with prenatal CHD were included in the analysis. TOP was recommended in 71 cases (48.6%). GA at diagnosis did not differ between groups when analyzed continuously; however, categorical GA showed significant differences, with earlier diagnoses more frequent among TOP-recommended cases. Cardiac severity distribution differed significantly between groups. In multivariable analysis, GA at diagnosis and cardiac severity were independently associated with TOP recommendation. Compared with <20 weeks, diagnosis at 20–23+6 weeks (OR 17.96, 95% CI 3.50–92.22) and ≥24 weeks (OR 3.92, 95% CI 1.53–10.06) increased the odds of TOP recommendation. Relative to high severity, moderate (OR 0.23, 95% CI 0.07–0.72) and low severity (OR 0.20, 95% CI 0.08–0.50) were associated with lower odds of TOP recommendation. Extracardiac anomalies, genetic findings, and FGR were not independently associated after adjustment. Similar patterns were observed in isolated CHD cases. **Conclusions:** In a multidisciplinary prenatal counseling setting, TOP recommendations after prenatal CHD diagnosis were primarily driven by cardiac severity and GA at diagnosis, rather than extracardiac or genetic findings alone.

## 1. Introduction

Congenital heart disease (CHD) is the most common group of major congenital anomalies, accounting for approximately one-third of all major birth defects worldwide [1]. With a pooled global prevalence of about 8–9 per 1000 live births, CHD affects an estimated 1.3–1.4 million newborns each year [2]. Advances in prenatal ultrasonography and fetal echocardiography have significantly improved the detection of CHD during pregnancy, enabling earlier diagnosis, refined anatomical characterization, and more detailed prognostic assessment. As a result, prenatal identification of CHD has become a key element of perinatal care, supporting counseling and planning of delivery and postnatal management.

Beyond its implications for postnatal treatment, a prenatal diagnosis of CHD introduces complex clinical, ethical, and psychosocial challenges for families and clinicians. Decisions regarding continuation or termination of pregnancy are influenced by multiple factors, including cardiac lesion severity, anticipated surgical complexity, long-term survival and quality of life, gestational age at diagnosis, and the presence of associated extracardiac or genetic anomalies [3,4]. Consequently, prenatal counseling after a CHD diagnosis extends beyond anatomical findings and often involves multidisciplinary clinical judgment.

Previous studies have consistently shown that cardiac severity plays a central role in parental decision-making and pregnancy outcomes following prenatal CHD diagnosis [5]. However, most available data focus on termination rates or parental choices rather than on recommendations made by clinicians within multidisciplinary decision-making processes [6]. In addition, the impact of gestational age at diagnosis, extracardiac findings, and genetic evaluation on termination recommendations is reported inconsistently across studies and appears to vary between settings [7,8]. These differences likely reflect variation in legal regulations, availability of postnatal surgical care, and local counseling practices.

In Turkey, prenatal CHD counseling is typically carried out in multidisciplinary fetal councils involving specialists from perinatology, pediatric cardiology, neonatology, and pediatric cardiovascular surgery [9]. Despite the routine use of this model in clinical practice, limited data are available on the factors underlying termination of pregnancy (TOP) recommendations made by such councils [10]. Understanding how clinical information is considered during this process may support more consistent prenatal counseling. In Turkey, termination of pregnancy for maternal request is legally permitted up to 10 weeks of gestation. However, in the presence of severe fetal anomalies or maternal indications, termination may be performed at later gestational ages following approval by a multidisciplinary medical board, and no strict upper gestational age limit is defined in the legislation. In clinical practice, termination at advanced gestational ages requires more extensive multidisciplinary evaluation and is subject to increased ethical and procedural considerations.

The aim of this study was to evaluate clinical factors associated with TOP recommendation following a prenatal diagnosis of CHD in a tertiary referral center, with particular attention to gestational age at diagnosis, cardiac severity, extracardiac anomalies, and genetic findings. By focusing on council-based recommendations rather than pregnancy outcomes alone, this study aims to describe clinician-driven decision-making within a multidisciplinary prenatal counseling model.

## 2. Materials and Methods

### 2.1. Study Design and Population

This retrospective cohort study was conducted at a tertiary referral center (Izmir City Hospital, Turkey) and included all pregnancies with a prenatal diagnosis of congenital heart disease (CHD), confirmed by detailed fetal echocardiography, that were discussed in the multidisciplinary fetal council between October 2023 and December 2025. This retrospective design was chosen due to ethical and practical constraints in evaluating real-world decision-making regarding termination of pregnancy without influencing patient management. The hospital is a tertiary referral center located in the western region of Turkey and receives pregnant women from a wide geographic area, including both the local region and surrounding provinces. As a public institution operating under the national health insurance system, it serves a broad and heterogeneous population, including patients from diverse socioeconomic backgrounds.

The fetal council is a structured multidisciplinary meeting involving perinatology, pediatric cardiology, medical genetics, neonatology, and, when indicated, pediatric cardiovascular surgery. Each case was reviewed using the complete fetal echocardiography report and comprehensive fetal anatomic ultrasound assessment by experienced maternal–fetal medicine specialists and pediatric cardiologists. Parents received standardized counseling addressing expected postnatal management options, potential surgical or interventional requirements, and overall prognosis, including possible long-term morbidity in severe cases and uncertainty in borderline lesions.

Genetic counseling was offered in collaboration with the medical genetics department. Counseling included discussion of the association between complex CHD and chromosomal/genetic conditions, available diagnostic options, and the scope and limitations of prenatal testing. When clinically indicated, invasive prenatal genetic testing was offered following genetic counseling and performed after parental consent, in accordance with routine institutional practice. In fetuses with structural anomalies, conventional karyotyping and chromosomal microarray analysis were routinely offered as first-line diagnostic tests and were provided within the standard healthcare coverage. Whole-exome sequencing (WES) was considered in selected cases with normal karyotype and microarray results, particularly when a monogenic etiology was suspected; however, this analysis required additional parental consent and out-of-pocket payment.

Following multidisciplinary evaluation, the council issued a medical recommendation regarding pregnancy continuation or TOP. The primary endpoint of this study was TOP recommendation by the fetal council (yes/no), documented in council records. In cases where TOP was recommended, the recommendation was presented to the parents, and the final decision regarding acceptance of the recommendation remained with the family. When TOP was not recommended, the council advised continuation of pregnancy and appropriate postnatal management.

Eligible cases were pregnancies with a confirmed prenatal diagnosis of congenital heart disease by fetal echocardiography, documented evaluation and recommendation by the multidisciplinary fetal council, and sufficient prenatal data to allow assignment of a primary cardiac diagnosis and disease severity. Multiple gestations and cases with missing key data precluding reliable cardiac classification were excluded. Because of the retrospective study design, no a priori sample size calculation was performed; instead, all eligible cases discussed during the predefined study period were included in the analysis.

### 2.2. Data Collection

Maternal demographic and obstetric characteristics (maternal age, gravidity, parity), gestational age at diagnosis, gestational age at council discussion or decision, fetal echocardiographic diagnosis, genetic evaluation status and documented results, and fetal council recommendation were retrospectively extracted from electronic medical records and council documentation. Associated extracardiac findings identified on prenatal ultrasound were recorded and categorized according to organ system involvement as central nervous system anomalies, skeletal or extremity anomalies, renal or gastrointestinal anomalies, soft markers, hydrops fetalis or cystic hygroma, and multiple anomalies. As all cases were evaluated and documented within a structured multidisciplinary fetal council using standardized clinical reporting, the quality and completeness of medical records were considered comparable between women who terminated the pregnancy and those who continued the gestation.

### 2.3. Cardiac Abnormality Diagnosis and Severity Classification

Fetal cardiac abnormality diagnoses were recorded as specific congenital heart disease (CHD) types based on detailed prenatal echocardiography. Each fetus was assigned a single primary cardiac diagnosis according to the dominant lesion identified at prenatal assessment. This approach mirrors routine prenatal cardiology practice, where defects are defined by their primary diagnostic entities rather than grouped categories.

The recorded CHD types included hypoplastic left heart syndrome, double outlet right ventricle, atrioventricular septal defect (including unbalanced forms), ventricular septal defect, coarctation of the aorta or interrupted aortic arch, transposition of the great arteries, congenitally corrected transposition of the great arteries, tetralogy of Fallot, tricuspid atresia, truncus arteriosus, Ebstein anomaly, persistent left superior vena cava, pulmonary stenosis or atresia, heterotaxy or isomerism, vascular ring, isolated tricuspid regurgitation, and fetal arrhythmia.

Cardiac severity was categorized into three strata (low, moderate, and high) reflecting anticipated hemodynamic burden, surgical complexity, and expected long-term outcome. Severity stratification was based on the fetal cardiovascular disease severity scale developed by Davey et al., a seven-level prenatal severity scale for structural congenital heart disease [11]. In the original scale, severity assessment is based on the anatomical complexity of the lesion, the anticipated need and number of postnatal interventions, the likelihood of biventricular repair versus single-ventricle palliation, and the overall expected prognosis. Genetic/chromosomal abnormalities and extracardiac anomalies were not incorporated into cardiac severity grading, in accordance with the original design of the Davey scale, and were therefore analyzed separately in the present study. In the current cohort, the seven-level scale was pragmatically grouped into clinically interpretable categories as low (grades 1–2), moderate (grades 3–4), and high (grades 5–7) to facilitate statistical analysis and reflect real-world prenatal counseling practice, in which risk communication is typically framed using broad severity categories rather than individual numeric scores. Severity classification was assigned based on prenatal echocardiographic findings during multidisciplinary fetal council evaluation by maternal–fetal medicine specialists and pediatric cardiologists before statistical analysis. The classification was determined by consensus during council review and reflected the clinical interpretation used in routine prenatal counseling. Because the classification was part of routine clinical evaluation, reviewers were not blinded to the clinical context of the cases.

### 2.4. Statistical Analysis

Statistical analyses were performed using IBM SPSS Statistics version 26.0. Continuous variables were tested for normality using the Kolmogorov–Smirnov test and are presented as mean ± standard deviation or median (minimum–maximum), as appropriate. Categorical variables are presented as number (%). Comparisons between cases with and without a termination of pregnancy (TOP) recommendation were performed using the Mann–Whitney U test for continuous variables and the χ^2^ test for categorical variables, as appropriate. Gestational age at diagnosis was analyzed both as a continuous variable and in predefined categories (<20 weeks, 20–23+6 weeks, and ≥24 weeks). Variables associated with TOP recommendation in univariable analyses and clinically relevant factors (gestational age at diagnosis, genetic evaluation results, cardiac severity, extracardiac anomalies, and fetal growth restriction) were included in a multivariable regression model to adjust for potential confounding. Due to the relatively high prevalence of the outcome (>10%), a Poisson regression model with robust variance estimation (sandwich estimator) was additionally performed to estimate relative risks (RR), as odds ratios (OR) may overestimate effect sizes in such settings. TOP recommendation (yes/no) was used as the dependent variable. Odds ratios (ORs) with 95% confidence intervals (CIs) were calculated. Model fit was assessed using −2 log likelihood and pseudo-R^2^ statistics. A subgroup analysis was performed among fetuses with isolated congenital heart disease, defined as the absence of extracardiac structural anomalies. A two-sided *p* value < 0.05 was considered statistically significant.

### 2.5. Ethical Approval

The study protocol was approved by the local non-interventional clinical research ethics committee (Approval no: 2026/180). Due to the retrospective nature of the study, the requirement for informed consent was waived. All data were anonymized prior to analysis, and the study was conducted in accordance with the Declaration of Helsinki. The authors declare no conflicts of interest.

## 3. Results

**Figure 1:**
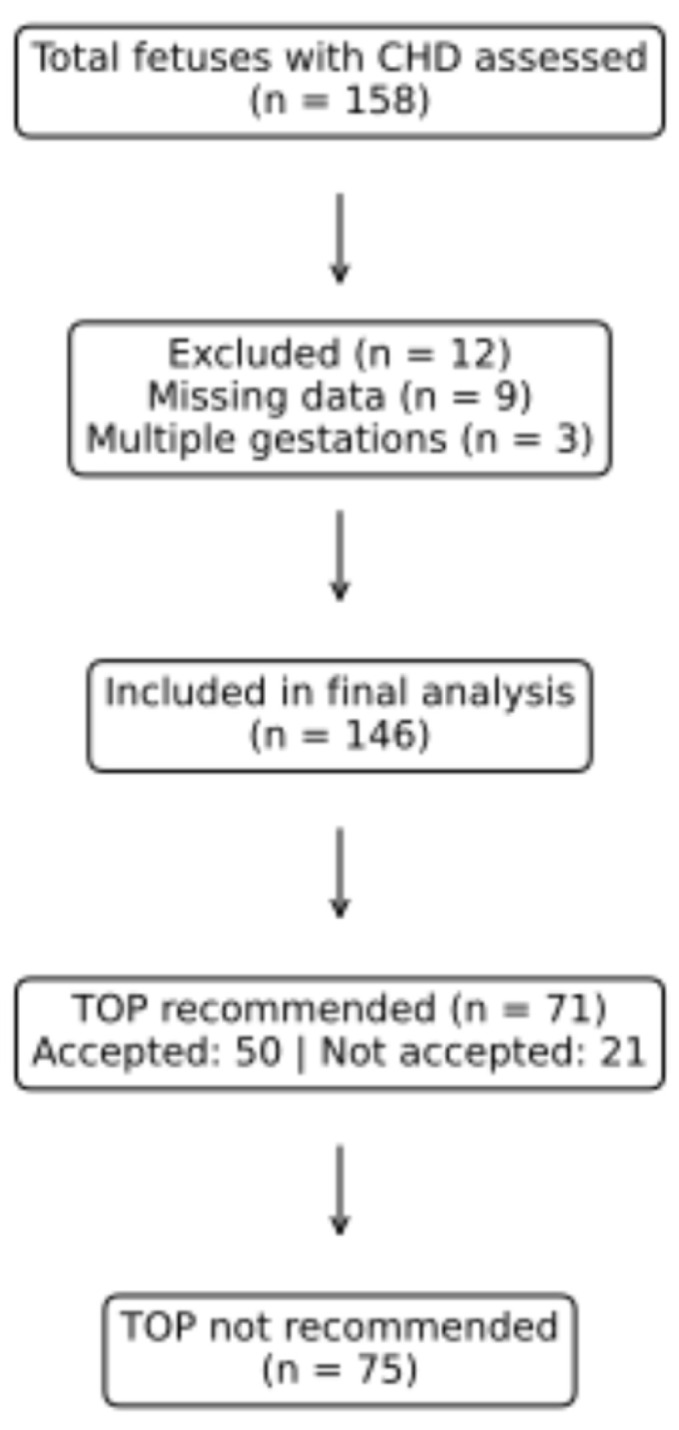
Flow diagram of study population selection and pregnancy outcomes.

A total of 158 fetuses with congenital heart disease were initially assessed. After applying predefined inclusion and exclusion criteria, 146 cases were included in the final analysis. Twelve cases were excluded due to missing key clinical data (*n* = 9) that precluded reliable cardiac classification or documentation of the multidisciplinary fetal council recommendation, and multiple gestations (*n* = 3). Termination was recommended in 71 cases (48.6%), whereas no termination recommendation was made in 75 cases (51.4%). Among the cases in which termination of pregnancy was recommended by the fetal council, 50 families (70.4%) accepted the recommendation and underwent termination, whereas 21 families (29.6%) chose to continue the pregnancy despite the recommendation (Figure 1). Maternal demographic characteristics, including maternal age, gravidity, parity, number of living children, and history of abortion, were similar between cases with and without a termination recommendation and are summarized in Table 1. When gestational age at diagnosis was analyzed as a continuous variable, no significant difference was observed between groups. However, when gestational age was analyzed categorically, a significant difference between groups was observed. Diagnoses established before 20 gestational weeks were significantly more frequent among cases with a termination recommendation, whereas diagnoses made at ≥24 gestational weeks were more common among cases without a termination recommendation.

The distribution of cardiac severity differed significantly between groups (Table 2). Termination recommendation was more frequent in fetuses with moderate and high cardiac severity, whereas low-severity anomalies were predominantly managed without a termination recommendation. None of the associated fetal anomalies (including hydrops or cystic hygroma, skeletal or limb anomalies, renal or gastrointestinal anomalies, multiple malformations, isolated soft markers, or central nervous system anomalies) showed a significant association with termination recommendation when evaluated individually. Similarly, fetal growth restriction was observed at comparable rates in cases with and without a termination recommendation. Although invasive genetic testing was performed more frequently in cases for whom termination was recommended, the overall rate of invasive testing did not differ significantly between groups. Invasive prenatal genetic testing was performed in 48 of 146 cases (32.9%). Among the tested fetuses, normal genetic findings were observed in 26 cases (54.2%), while major chromosomal or pathogenic abnormalities were identified in 22 cases (45.8%). Genetic evaluation was not performed or could not be completed in the remaining 98 cases (67.1%).

Findings from the binary logistic regression analyses are presented in Table 3. In these analyses, gestational age at diagnosis emerged as the strongest independent factor associated with termination recommendation. Using diagnoses established before 20 gestational weeks as the reference category, diagnoses made at 20–23+6 gestational weeks and at ≥24 gestational weeks were associated with significantly higher odds of termination recommendation. Cardiac severity was also independently associated with termination recommendation; compared with high cardiac severity, moderate and low severity categories were associated with significantly lower odds of termination recommendation. In contrast, extracardiac anomalies, genetic test results, and fetal growth restriction were not independently associated with termination recommendation after adjustment. The model demonstrated adequate explanatory power (Nagelkerke R^2^ = 0.365).

In the subgroup analysis of 75 fetuses with isolated CHD, termination recommendation was significantly associated with earlier gestational age at diagnosis and high cardiac severity (Table 4). In this subgroup, fetal growth restriction and patterns of genetic evaluation did not differ significantly between cases with and without a termination recommendation. Invasive genetic testing was performed in 23 isolated cases (30.7%); among these, normal genetic results were identified in 17 cases, whereas major chromosomal or pathogenic anomalies were detected in 6 cases.

The distribution of CHD types according to cardiac severity categories is shown in Table 5. The representative examples of CHD lesions according to severity categories are provided in Supplementary Table S1. Nearly half of the study population (47.3%) was classified as high severity, followed by moderate (36.3%) and low (16.4%) severity categories, reflecting the broad spectrum of cardiac anomalies evaluated in this cohort.

## 4. Discussion

A prenatal diagnosis of CHD has implications not only for fetal prognosis but also for complex clinical and ethical decision-making processes involving both families and clinicians. In this study, we analyzed clinical factors associated with TOP recommendation in pregnancies diagnosed with CHD in the prenatal period and evaluated by a multidisciplinary perinatology council. Our findings demonstrate that gestational age at diagnosis and cardiac severity were independently associated with TOP recommendation, whereas extracardiac anomalies, genetic findings, and fetal growth restriction were not identified as independent factors. These results suggest that TOP recommendation in prenatal counseling is shaped by a comprehensive clinical assessment rather than reliance on a single diagnostic parameter. In our cohort, approximately 70% of families accepted the recommendation for termination, highlighting the substantial influence of multidisciplinary counseling on parental decision-making, while also indicating that final decisions are not solely determined by clinical recommendations.

CHD diagnosed in the prenatal period presents a broad clinical spectrum in terms of postnatal treatment requirements, surgical complexity, long-term survival, and quality of life. In the literature, lesions such as hypoplastic left heart syndrome, complex single-ventricle physiology, severe conotruncal anomalies, and isomerism syndromes are among the conditions for which TOP is most frequently discussed during prenatal counseling in many centers [12,13]. These lesions are generally associated with high surgical morbidity, the need for staged surgical interventions, and uncertainty regarding long-term outcomes [14]. In contrast, some cardiac anomalies of moderate severity are often regarded as a “gray zone” in the prenatal period. In a prospective study by Gowda et al., counseling approaches for moderately severe lesions were shown to vary substantially depending on center experience and postnatal surgical outcomes [15]. Isolated ventricular septal defects, mild outflow tract obstructions, or cardiac anomalies with limited hemodynamic impact typically constitute cases in which TOP is not recommended and continuation of pregnancy is preferred [16]. This wide clinical spectrum suggests that TOP decisions are influenced not only by lesion type but also by the expected clinical course and anticipated postnatal management.

The role of cardiac severity as a determinant of TOP recommendation represents one of the most consistent and reliable findings of our study. We applied the seven-level fetal cardiovascular disease severity scale defined by Davey et al., which was pragmatically reclassified into three categories (low, moderate, and high severity) to enhance clinical applicability [11]. This approach facilitates clearer and more standardized risk communication during prenatal counseling. Our findings show that the likelihood of a TOP recommendation increases significantly with greater cardiac severity, underscoring the central role of disease burden in clinician-driven decision-making. While much of the existing literature has focused on the association between cardiac severity and parental decision-making, fewer studies have specifically addressed how clinician recommendations regarding TOP vary according to disease severity [4,5]. In this context, the study by Calzada-Lozada et al. demonstrated that increasing lesion severity was associated with more frequent discussion of TOP and palliative care options during fetal cardiac counseling [17]. Our findings are consistent with these observations, as increasing cardiac severity was independently associated with a higher likelihood of TOP recommendation in our cohort.

Gestational age at diagnosis has also been consistently reported as an important factor influencing TOP-related decisions. Montaguti et al. reported that diagnoses established in the early second trimester are frequently associated with prognostic uncertainty, which in turn leads to a more cautious approach to counseling [5]. Conversely, several studies have reported higher TOP rates among cases diagnosed at later gestational ages [18]. In our cohort, when gestational age at diagnosis was analyzed categorically, diagnoses established at ≥20 weeks’ gestation were independently associated with a higher likelihood of TOP recommendation compared with the reference group diagnosed before 20 weeks. This finding does not reflect a direct protective effect of early diagnosis but rather suggests that differences in diagnostic timing are associated with variations in clinical presentation and diagnostic clarity. While cases diagnosed earlier in pregnancy tend to represent a more heterogeneous group, those diagnosed at later gestational ages are more likely to exhibit well-defined anatomical features and clearer prognostic implications.

Interpretation of genetic evaluation findings requires consideration of the nature of prenatal diagnosis and parental expectations. In clinical practice, families considering TOP are more likely to undergo prenatal genetic testing, whereas those planning to continue the pregnancy may prefer to defer genetic evaluation to the postnatal period [19]. This observation is in line with the findings of Ngan et al., indicating that parental perceptions of disability and expected quality of life play a greater role in decisions about prenatal genetic testing and TOP than genetic results alone [19]. Accordingly, the higher frequency of genetic testing among cases with a TOP recommendation in our study is more likely to reflect selection within the counseling process rather than a direct causal role of genetic findings. Major genetic anomalies represent situations in which TOP is routinely discussed during prenatal CHD counseling and considered together with cardiac severity and expected quality of life. In addition, the requirement for out-of-pocket payment for WES in our setting may have further influenced testing uptake and introduced potential socioeconomic selection bias. Given that invasive genetic testing was performed in a limited proportion of cases, the lack of a significant association between genetic findings and termination decisions should be interpreted with caution; however, genetic findings were not identified as an independent predictor in multivariable analysis, suggesting a limited independent impact on termination decisions within this cohort, where clinical factors such as cardiac severity and gestational age appear to play a more dominant role.

Nevertheless, TOP recommendations following a prenatal diagnosis of CHD may vary considerably across countries despite similar clinical parameters. These differences are closely linked to variations in legal frameworks, healthcare system capacity, and prenatal counseling practices. Studies from North America and Western Europe have shown that TOP rates do not necessarily increase in parallel with rising prenatal detection rates [20,21]. In large population-based studies from the United States, prenatal diagnosis rates have increased substantially over time, while TOP rates have remained relatively stable [21]. In these cohorts, TOP decisions were more strongly associated with syndromic conditions or significant extracardiac morbidity than with cardiac lesion severity alone. Even among cases with functional single-ventricle physiology, TOP rates were relatively low in centers offering advanced postnatal surgical and intensive care support [20].

By contrast, Montaguti et al. reported that TOP decisions in their Italian cohort were mainly related to surgical complexity and the expected postnatal course [5]. Although chromosomal abnormalities were considered, surgical complexity and the anticipated postnatal course appeared to play a greater role in family decision-making [5]. This suggests that, in well-resourced healthcare systems, decisions are influenced not only by the diagnosis itself but also by expectations regarding postnatal management. Studies from Taiwan have highlighted the central role of counseling models in TOP decisions following a prenatal CHD diagnosis [3]. In these reports, the strongest predictors of TOP included surgical requirements, single-ventricle physiology, and expectations of staged surgical repair. Importantly, families who received prenatal counseling from pediatric cardiologists were significantly more likely to continue the pregnancy [3]. Taken together, these findings suggest that decisions after a prenatal diagnosis of CHD depend not only on lesion severity but also on the structure and delivery of prenatal counseling. In our study, gestational age at diagnosis and cardiac severity emerged as the strongest independent determinants of TOP recommendation. The absence of independent associations for extracardiac anomalies, fetal growth restriction, and genetic findings in multivariable analyses suggests that, in Turkey, TOP recommendations are based on an overall clinical assessment within a multidisciplinary fetal council rather than on isolated findings. The strong association with gestational age highlights the role of legal limits and local clinical practice in shaping prenatal counseling. In cases diagnosed beyond 24 weeks of gestation, termination decisions require multidisciplinary board approval rather than being determined by a strict legal threshold. In this setting, decisions are mainly driven by clinical severity and overall prognostic assessment within the fetal council. In this context, our findings suggest a decision-making approach in Turkey that differs from both the surgery-focused models commonly reported in Western countries and the counseling-centered approaches described in East Asian studies. Importantly, similar findings obtained with Poisson regression models using robust variance estimation support that the observed associations are not solely dependent on the choice of statistical model and remain clinically consistent.

Several limitations of this study should be acknowledged. The retrospective design and relatively limited sample size may have reduced statistical power, particularly in subgroup analyses. In addition, some estimates were associated with wide confidence intervals, likely reflecting limited statistical power in certain subgroups. However, the consistency of findings across both logistic and Poisson regression models with robust variance estimation supports the robustness of the observed associations. Additionally, the study was conducted in a tertiary referral center, resulting in a more selected patient population. While this may limit the generalizability of our findings, it also strengthens the clinical relevance of the study by focusing on cases in which TOP decisions are most complex and challenging.

In conclusion, this study shows that TOP recommendations following a prenatal diagnosis of congenital heart disease are influenced by disease characteristics, timing of diagnosis, prenatal counseling, and the healthcare system context. Our findings highlight the central role of cardiac severity and gestational age at diagnosis, while indicating that genetic and extracardiac findings alone do not independently determine recommendations. In line with the international literature, TOP decisions appear to be sensitive not only to medical parameters but also to the structure and delivery of prenatal counseling. From this perspective, our study highlights the importance of standardized and multidisciplinary approaches in prenatal CHD counseling.

## Figures and Tables

**Table 1:** Demographic characteristics of the study population according to termination recommendation.

Variable	Termination Not Recommended	Termination Recommended	*p* Value
Maternal age (years), median (min–max)	29 (17–46)	29 (19–45)	0.498
Gravida, median (min–max)	2 (1–7)	2 (1–7)	0.833
Parity, median (min–max)	1 (0–4)	1 (0–5)	0.512
Living children, median (min–max)	1 (0–4)	1 (0–5)	0.538
Abortions, median (min–max)	0 (0–4)	0 (0–4)	0.596
Gestational age at diagnosis (days), median (min–max)	175 (97–268)	179 (84–259)	0.398
Gestational age at diagnosis (weeks + days)	25+0 (13+6–38+2)	25+4 (12+0–37+0)	0.398
Gestational age category, *n* (%)			<0.001
GA < 20 weeks, *n* (%)	2 (10.5)	17 (89.5)	
GA 20–23+6 weeks, *n* (%)	20 (45.5)	24 (54.5)	
GA ≥ 24 weeks, *n* (%)	53 (63.9)	30 (36.1)	

Data are presented as median (range) or number (%), as appropriate. *p* values represent overall comparisons between groups using the chi-square test.

**Table 2:** Cardiac severity, associated fetal conditions, and genetic characteristics stratified by termination recommendation.

Variable	Termination Not Recommended *n* (%)	Termination Recommended *n* (%)	Total *n* (%)	*p*
Cardiac severity				
Low	10 (41.7)	14 (58.3)	24 (16.4)	0.414
Moderate	18 (33.3)	36 (66.7)	54 (37.0)	0.009
High	43 (63.2)	25 (36.8)	68 (46.6)	0.022
Associated fetal conditions *				
Hydrops/cystic hygroma	5 (3.4)	3 (2.1)	8 (5.5)	>0.05
Skeletal/limb anomalies	8 (5.5)	6 (4.1)	14 (9.6)	
Multiple malformations/syndromic appearance	4 (2.7)	8 (5.5)	12 (8.2)	
Renal/gastrointestinal anomalies	4 (2.7)	7 (4.8)	11 (7.5)	
Only soft marker	7 (4.8)	4 (2.7)	11 (7.5)	
CNS anomalies	4 (2.7)	11 (7.5)	15 (10.3)	
Fetal growth status				
IUGR present	21 (14.4)	18 (12.3)	39 (26.7)	0.631
IUGR absent	50 (34.2)	57 (39.0)	107 (73.3)	0.499
Genetic evaluation **, *n* (%) ***				>0.05
Invasive genetic testing performed	22 (45.8)	26 (54.2)	48 (32.9)	
Genetic testing not performed	49 (50.0)	49 (50.0)	98 (67.1)	

* Each fetus was assigned to the most clinically relevant associated fetal condition. ** Invasive prenatal genetic testing was performed in 48 of 146 cases (32.9%). Among the tested fetuses, normal genetic findings were observed in 26 cases (54.2%), while major chromosomal or pathogenic abnormalities were identified in 22 cases (45.8%). Genetic evaluation was not performed or could not be completed in the remaining 98 cases (67.1%). These values represent the distribution of genetic results within the tested subgroup and are independent of the termination-based stratification presented in Table 2. *** Percentages are calculated within each subgroup and represent within-group distributions.

**Table 3:** Binary logistic regression analysis of factors associated with termination recommendation.

Variable	OR	95% CI	*p* Value
Extracardiac anomalies			0.293
Hydrops/cystic hygroma	0.26	0.06–1.03	0.055
Skeletal/limb anomalies	0.18	0.02–1.76	0.139
Multiple malformations/syndromic appearance	0.29	0.05–1.65	0.165
Renal/gastrointestinal anomalies	0.72	0.10–5.30	0.751
Only soft marker	1.15	0.16–8.13	0.889
CNS anomalies	0.25	0.04–1.76	0.165
Cardiac severity (3 categories)			0.001
Moderate vs. High	0.23	0.07–0.72	0.012
Low vs. High	0.20	0.08–0.50	0.001
Genetic result			0.713
Major anomaly vs. no test	0.77	0.27–2.20	0.621
Normal vs. no test	0.54	0.12–2.37	0.411
IUGR	0.49	0.20–1.21	0.123
Gestational age at diagnosis			<0.001
20–23+6 weeks vs. <20 weeks	17.96	3.50–92.22	0.001
≥24 weeks vs. <20 weeks	3.92	1.53–10.06	0.004

Binary logistic regression analysis was performed with termination recommendation as the dependent variable. Reference categories were defined as follows: High severity for cardiac severity, no genetic testing for genetic result, absence of extracardiac anomalies for anomaly categories, absence of IUGR for fetal growth status, and <20 weeks for gestational age at diagnosis.

**Table 4:** Comparison of isolated congenital heart disease cases according to termination of pregnancy recommendation.

Variable	TOP Not Recommended *n* (%)	TOP Recommended *n* (%)	Total *n* (%)	*p* Value
Maternal age (years), median (range)	30 (17–46)	28 (19–42)	—	0.778
Gestational age at diagnosis (weeks), median (range)	28+6 (19+0–38+2)	22+2 (16+1–33+6)	—	0.001
Gestational age at diagnosis, *n* (%)				0.003
<20 weeks	1 (2.1)	3 (11.1)	4 (5.3)	
20–23+6 weeks	15 (31.3)	13 (48.1)	28 (37.3)	
≥24 weeks	32 (66.7)	11 (40.7)	43 (57.3)	
IUGR, *n* (%)				0.386
Absent	36 (48.0)	23 (30.7)	59 (78.7)	
Present	12 (16.0)	4 (5.3)	16 (21.3)	
Cardiac severity (isolated CHD), *n* (%)				<0.001
Low–Moderate *	33 (44.0)	5 (6.7)	38 (50.7)	
High	15 (20.0)	22 (29.3)	37 (49.3)	
Genetic evaluation, *n* (%) **				0.069
Invasive genetic testing performed	11 (14.7)	12 (16.0)	23 (30.7)	
Genetic testing not performed	37 (49.3)	15 (20.0)	52 (69.3)	
Total	48 (64.0)	27 (36.0)	75 (100)	

* For the isolated CHD subgroup analysis, low and moderate severity categories were combined due to limited sample size. ** In the isolated congenital heart disease subgroup, invasive genetic testing was performed in 23 of 75 cases (30.7%). Among those tested, 17 (73.9%) had normal genetic results, and 6 (26.1%) had major chromosomal or pathogenic anomalies. Isolated congenital heart disease was defined as the absence of extracardiac structural anomalies. Data are presented as median [range] or number (%).

**Table 5:** Distribution of congenital heart disease types by cardiac severity category.

Cardiac Severity Category	Total *n* (%)	CHD Types Within Category, *n* (%)
High (3)	69 (47.3)	HLHS 13 (8.9); Unbalanced AVSD 11 (7.5); DORV 9 (6.2); Truncus arteriosus 8 (5.5); Others ≤ 4.8
Moderate (2)	53 (36.3)	CoA/IAA 13 (8.9); DORV 10 (6.8); TGA 6 (4.1); TOF 6 (4.1); Others ≤ 3.4
Low (1)	24 (16.4)	VSD 15 (10.3); LSVC 5 (3.4); Others ≤ 1.4

Values are presented as number (%). Percentages refer to the total study population (*n* = 146). CHD types are listed in descending order of frequency within each severity category.

## Data Availability

Data are not publicly available due to ethical restrictions. Further inquiries can be directed to the corresponding author.

## Supplementary Materials

The following supporting information can be downloaded at: https://www.mdpi.com/article/10.3390/jcm15082838/s1, Table S1: Representative examples of CHD lesions according to grouped Davey severity categories.

## Abbreviations

The following abbreviations are used in this manuscript:

AVSD	Atrioventricular septal defect
CHD	Congenital heart disease
CI	Confidence interval
CNS	Central nervous system
CoA	Coarctation of the aorta
DORV	Double outlet right ventricle
FGR	Fetal growth restriction
GA	Gestational age
HLHS	Hypoplastic left heart syndrome
IAA	Interrupted aortic arch
IUGR	Intrauterine growth restriction
LSVC	Left superior vena cava
OR	Odds ratio
TOP	Termination of pregnancy
TGA	Transposition of the great arteries
TOF	Tetralogy of Fallot
WES	Whole-exome sequencing
