# Supplementary material for: Clinical Factors Associated with Termination of Pregnancy Recommendations Following Prenatal Diagnosis of Congenital Heart Disease: A Multidisciplinary Council-Based Study
Source: J Clin Med. 2026 Apr 9;15(8):2838. doi: 10.3390/jcm15082838 (PMC13116180; doi:10.3390/jcm15082838)
Supplement: Supplementary file 1 [file jcm-15-02838-s001.zip › jcm-4200066-supplementary.pdf]

Supplementary Table S1. Representative examples of CHD lesions according to grouped Davey severity categories.

Grouped severity category used in this study	Corresponding Davey grades	Representative examples based on the Davey fetal cardiovascular disease severity framework
**Low severity**	**Grades 1–2**	Isolated small/midmuscular ventricular septal defect; isolated echogenic intracardiac focus/otherwise structurally normal heart; isolated premature atrial contractions with otherwise normal cardiac structure; mild cardiovascular abnormalities that may only require follow-up or possible later intervention
**Moderate severity**	**Grades 3–4**	Tetralogy of Fallot with mild pulmonary stenosis; transposition of the great arteries with intact ventricular septum; balanced complete atrioventricular septal defect without significant AV valve regurgitation; coarctation of the aorta with preserved ventricular adequacy; interrupted aortic arch with VSD and mild subaortic narrowing; critical aortic stenosis with preserved left ventricular size/function (borderline/intermediate example)
**High severity**	**Grades 5–7**	Hypoplastic left heart syndrome; heterotaxy with single-ventricle physiology; tricuspid atresia; pulmonary atresia with intact ventricular septum and marked right ventricular hypoplasia; truncus arteriosus with major associated valve abnormality; Ebstein anomaly with severe tricuspid regurgitation and hydrops; complex conotruncal disease or single-ventricle pathways expected to require staged palliation or associated with poor prognosis

Footnote:
This table provides representative examples derived from the conceptual framework and example cases presented in the Davey fetal cardiovascular disease severity scale. Because the original Davey scale is based on overall prenatal prognostic assessment rather than a fixed lesion-to-category list, some lesions may vary in severity assignment according to anatomic details, expected postnatal management, and anticipated prognosis.
